# Patients' Experience of Winter Depression and Light Room Treatment

**DOI:** 10.1155/2017/6867957

**Published:** 2017-02-15

**Authors:** Cecilia Rastad, Lennart Wetterberg, Cathrin Martin

**Affiliations:** ^1^Department of Neuroscience, Uppsala University, Uppsala, Sweden; ^2^Center for Clinical Research Dalarna (CKF), Falun, Sweden; ^3^Department of Clinical Neuroscience, Karolinska Institute, St. Göran, Stockholm, Sweden

## Abstract

*Background*. There is a need for more knowledge on the effects of light room treatment in patients with seasonal affective disorder and to explore patients' subjective experience of the disease and the treatment.* Methods*. This was a descriptive and explorative study applying qualitative content analysis. A purposeful sample of 18 psychiatric outpatients with a major depressive disorder with a seasonal pattern and a pretreatment score ≥12 on the 9-item Montgomery-Åsberg Depression self-rating scale was included (10 women and 8 men, aged 24–65 years). All patients had completed light room treatment (≥7/10 consecutive weekdays). Data was collected two weeks after treatment using a semistructured interview guide.* Results*. Patients described a clear seasonal pattern and a profound struggle to adapt to seasonal changes during the winter, including deterioration in sleep, daily rhythms, energy level, mood, activity, and cognitive functioning. Everyday life was affected with reduced work capacity, social withdrawal, and disturbed relations with family and friends. The light room treatment resulted in a radical and rapid improvement in all the major symptoms with only mild and transient side effects.* Discussion*. The results indicate that light room treatment is essential for some patients' ability to cope with seasonal affective disorder.

## 1. Introduction

The clinical features of seasonal affective disorder (SAD) were first described in 1984 in the classic study by Rosenthal et al. [1]. In the Diagnostic and Statistical Manual of Mental Disorders (DSM-5) [2], depression with a seasonal pattern is classified as a subtype of major depressive disorder or bipolar disorder. A diagnosis requires having these experiences for at least two years, a depression that regularly begins and ends during a specific season, no episodes of depression during the season in which the patient experiences normal mood (past two years), and more seasons with than seasons without depression over the lifetime of the illness. In this study, the concept winter depression is used interchangeably with SAD-winter type.

The prevalence of SAD varies between 1% and 2.4% in general population samples [3, 4]. A number of studies have shown that approximately 10–20% of the general population in northern countries report similar but less impairing symptoms [5, 6]. These persons suffer from subsyndromal SAD (winter fatigue) and not major depression [7]. Winter fatigue is associated with lower ratings of health-related quality of life [8, 9]. Reviews on the neurobiology of SAD are published elsewhere [10–13].

A nonpharmacological treatment for SAD is light therapy (LT) [1, 14]. Despite a number of studies over the years, the evidence for LT remains an unresolved issue. Some maintain that it is the first-line therapy for SAD [15, 16], but in Sweden it is not approved as an evidence-based treatment [17]. In a review of randomized placebo-controlled trials, eight studies of LT in SAD and two studies of LT in nonseasonal depression were included [18]. In all these studies, only light boxes were used and the treatment effects were restricted to short-term effects. In conclusion, the authors considered the evidence “not unequivocal” [18]. A recent randomized, double-blind, placebo- and sham-controlled trial of patients with nonseasonal depression (*n* = 122) reported that LT was an effective treatment both as a monotherapy and in combination with antidepressant medication [19]. In one study by Lam et al., LT and antidepressant medication in patients with SAD were equally effective in reducing depressed mood [20].

**Figure 1:**
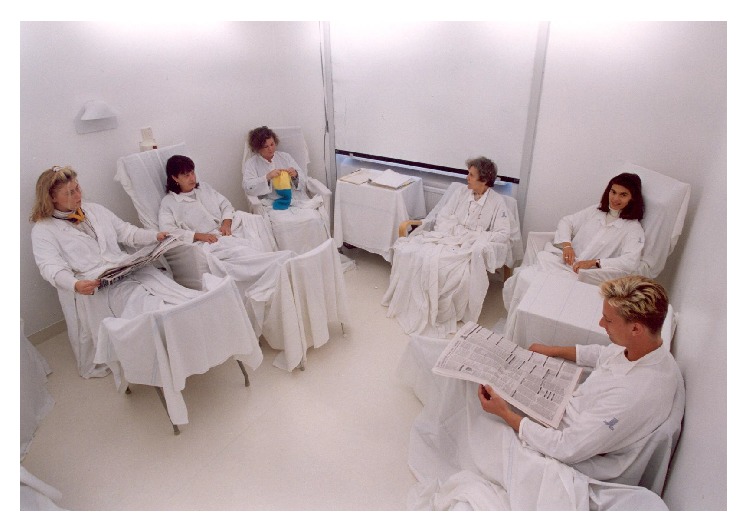
Illustration of light treatment in a room comparable to those used in the present study. Volunteers from the medical staff have in the photograph replaced the patients to keep the participants' privacy (by consent of the photographer Stig-Göran Nilsson).

Most clinical studies evaluating the effects of LT have used light boxes and portable devices used for treatment in patients' home [14]. In Swedish public health care, light therapy is also provided in light rooms (Figure 1). The light room was designed by professor Wetterberg and colleagues at the Karolinska Institute in Stockholm during the 1970s primarily for research of endocrine responses to light in humans [21]. This setting was further developed into light rooms for treatment studies in the 1980s and 1990s. The advantages of light rooms were improved control of treatment compliance and the ability to treat several patients at the same time and under similar light conditions [7, 22, 23]. Light rooms for research purposes of SAD were also introduced in Finland during the same time period [24].

There are some differences in the setting between treatment at home using a light box and treatment in a light room that is situated in a health care environment [7], which is why the results of clinical trials with light boxes cannot automatically be transferred to the light room settings [8, 22, 25, 26]. In a study with a factorial experimental design by Thalén and coworkers, the effects of light room treatment were compared in two diagnostic subgroups under the same light conditions [26]. The treatment effect in patients with SAD was larger compared to those with non-SAD, suggesting a more explicit effect of light room treatment in patients with SAD [26]. In a controlled clinical study by Rastad and coauthors, patients with SAD were randomized to light room treatment or a three-week waiting list followed by light room treatment [25]. A total of 54% in the experimental condition improved ≥50%, while no such improvement was seen in the control condition. After merging the two groups at the one-month follow-up, 83% had improved ≥50% and 64% were within the normal range in depression scores [25].

In a Cochrane review 2015, it was concluded that comparative evidence regarding LT versus other treatment options for preventing winter depression is limited [27]. The authors therefore recommend that the selection of treatment should be strongly based on patient preferences. What do we know about the patients' perspective on light treatment? One way to explore the subject of patient preferences is to use qualitative research designs. When this study was planned, there were no previous publications exploring the patients' subjective experience of light room treatment.

A starting point for the study was the biopsychosocial model [28]. This model, in addition to a biomedical model, includes a combination of biological, psychological, and social factors in order to obtain a broader description and understanding of the patients' perspective.

The aim of this study was to explore and to describe the patients' subjective experience of the disease seasonal affective disorder and the treatment in a light room.

## 2. Method

### 2.1. Design

This was a descriptive and explorative study applying qualitative content analysis in accordance with the procedures described by Graneheim and Lundman [29].

### 2.2. Participants and Procedure

A purposeful sample of 19 patients with seasonal affective disorder (SAD) referred to a psychiatric outpatient clinic in Stockholm was included. The clinic is situated at latitude 60°N. One patient left the study for personal reasons. Thus, the final sample consisted of 18 patients: 10 women and 8 men, aged 24–65 years (Md = 41 years) (Table 1).

Inclusion criteria were a history of major depressive disorder with a winter seasonal pattern (subtype) according to the diagnostic manual DSM-IV [30], ≥18 years of age, being able to participate in an interview conducted in Swedish, a pretreatment scoring ≥12 on the Montgomery-Åsberg Depression Rating Scale self-rating version (MADRS-S), and having completed treatment in the light room (≥7–10 consecutive weekdays). Four patients scheduled for preventative treatment were included (*N* = 4/18) and, for these patients, the inclusion criteria regarding depressed mood (MADRS-S) were not applied. Patients with a bipolar disorder, delusional disorder, severe personality, or eating disorder or with current abuse were excluded.

A registered nurse (RN) at the psychiatric outpatient clinic gave oral and written information about the project and asked possible informants about participation in the study. An experienced psychiatrist (LW) made the final inclusion of informants based on information from the referral, the patient record, and the total score on the pretreatment MADRS-S. The RN was responsible for the treatment in the light rooms and the registration of attendance at each session. The interviews (conducted by CR) were carried out during the two winter seasons (Oct–Feb) 2012-2013 and (Nov–March) 2013-2014. The self-report questionnaires were filled out during the first day and the last day of the treatment at the psychiatric outpatient clinic. The demographic background data were collected at the first day of the treatment. The two-week posttreatment questionnaires and a prepaid return envelope were sent by mail to the informant's home address at the time of the interview with a letter requesting the informants to fill out the form and return it by mail within a week.

Sampling was stopped when a preliminary evaluation suggested that adding more informants would not elicit any fundamentally new information not already provided by previous informants [31]. The Regional Ethical Review Board in Stockholm, Sweden, approved this study (Dnr 2012/1-31/3) before its start. The study was conducted in accordance with the Declaration of Helsinki (1964).

### 2.3. Measures

In this study, the 9-item patient-administered self-rating version of the MADRS (MADR-S) [32–34] was used. The items concern sadness, inner tension, reduced sleep and appetite, concentration difficulties, lassitude, inability to feel, pessimistic thought, and suicidal thought. Each item is scored between 0 (no symptoms) and 6 (severe symptoms). The total score ranges between 0 and 54.* Attrition*. The few single missing values (*N* = 2) on the pretreatment MADRS-S were imputed using the median value. There were no missing values on the posttreatment MADRS-S. The few single missing values (*N* = 3) in the two weeks after treatment MADRS-S were imputed with the value from the previous measurement (the posttreatment MADRS-S). There was a total of 4/18 missing MADRS-S at two weeks after treatment.

### 2.4. The Light Room Treatment

The light room treatment was provided at a psychiatric outpatient clinic in the western part of Stockholm. The walls in the light rooms were painted in white/light colors and the furniture was covered with white sheets. Patients rested comfortably in armchairs in small groups reading, talking, or just relaxing (Figure 1). Treatment was given daily for 2 h on weekdays (between 8 and 12 am) during the period from October to March. A minority of informants preferred shorter treatments between 1 and 2 h. Shaded fluorescent tubes with 6500 Kelvin, R_0_94 rendering average, reflecting the light on the ceiling and on the walls provided the configuration of the light source. Light intensity was 350 candela/m^2^ (1500 lux at eye level, measured 0.8 meters above the floor).

### 2.5. The Interview

A semistructured interview guide (The Interview Guide) was used during the telephone interviews (CR) covering the two areas of interest. Follow-up questions (e.g., “can you tell more” and “can you give an example”) were used to clarify or to go in more depth on the experiences related to the research objectives. A pilot interview was included in the analysis with only minor changes in the interview guide. The interviews lasted between 20 and 60 min (Md 33 min).

*The Interview Guide*

Light room treatment:

1. Is this the first time you have had light therapy or have you tried it before?
2. Can you explain in as much detail as possible how you experienced the treatment?
3. What is the best thing and worst thing about light therapy in your experience?
4. Do you remember any time in the past when you felt that you really benefited from light therapy?
5. Describe an occasion when you had a negative experience of the treatment.
6. Do you think your life and everyday activities are affected by the treatment?

Seasonal affective disorder:

(7) Can you describe how you feel affected by the different seasons of the year?
(8) Can you describe an occasion when you found winter depression was especially hard?
(9) Can you do anything yourself to affect how you feel and cope during the winter months?
(10) Do you have winter depression in your own opinion?

### 2.6. Data Analysis

The interviews were transcribed verbatim [35] and analyzed with qualitative content analysis [29]. First, all interviews were read repeatedly to obtain a general impression of the content. Second, the parts of the interviews related to the research objectives were marked, extracted, and labeled with a condensed description of the meaning (meaning unit). Third, the meaning units were divided into subcategories and categories. The division into subcategories and categories was repeated and rearranged several times by the first author in collaboration with the coauthors until agreement was obtained [29]. Through this process of reflection and discussion, the final themes, categories, and subcategories were agreed upon (Tables 2 and 3). For the purpose of illustration, citations in the form of condensed meaning units, that is, excluding repeats, were used in Results [29]. Interviews and content analysis were performed in Swedish. An experienced, bilingual translator translated the citations into English.

### 2.7. Trustworthiness

In order to increase credibility and transferability, we strived to capture a variety of experiences by heterogeneity among participants regarding sex, severity of depression, and previous experience of light room treatment. The rich variation in the data suggests that the sampling procedure was relevant. The presentation of the informants' background characteristics (Table 1) makes it possible for the reader to evaluate the extent to which the findings can be transferable to another sample. Trustworthiness was increased through dialogue with subsequent agreement between coresearchers (researcher triangulation) [29]. The clinical and research experience among the authors was considered of importance for the planning of the study and the analysis. The first author (CR) has more than 20 years of experience from clinical work as a registered physiotherapist in psychiatric outpatient health care and 9 years of clinical experience with light room treatment. The second author (LW) is a psychiatrist with extensive research experience and is the founder of the light room treatment. The third author's (CM) research area has been primarily in social sciences and qualitative method.

## 3. Results

### 3.1. Patients' Experience of Winter Depression

The analysis of patients' experience of seasonal affective disorder resulted in one main theme,* “struggling to adapt to the inevitable*,*”* three categories, and 19 subcategories (Table 2). Categories and subcategories are described below and illustrated with quotations. The numeral following each quotation refers to the informants. An overview of the informants is presented in Table 1.

#### 3.1.1. A Clear Seasonal Pattern

In the late summer or beginning of the fall, informants described that they* became aware of the seasonal shift in the form of a signal, a personal symptom that functioned as a marker* for worse times soon to come. It was described as a sudden recognition of a thought, a feeling, or a change in behavior. “It creeps up on you when it starts getting cold outside or when the nights start drawing in. Sometimes it just takes someone to say, ‘Oh, how lovely now that autumn is on the way' to make me think, ‘ugh, it's soon autumn.' I'm engulfed in a feeling of unease for a few moments, a feeling which then creeps up on me more and more.” (3)

It is the experience of a clear difference in feelings, thoughts, and behavior between the summer season and the winter season over longer periods that constitute the pattern. To* discover the pattern* therefore takes several years. “I manage to do much more in the summer, especially after work. I have more energy. That is the most obvious difference. Things like meeting friends, taking exercise or just being outdoors and going for a walk and enjoying the light of an evening. Those are things I don't do in the winter.” (13) The depressions vary over the years. “I've felt the change of season for some thirty years. But I've not been depressed for thirty years; rather, it's been the last eight to ten years that I've found every autumn really difficult.” (2) Some of the informants experienced seasonal depressions that continued for 1 or 2 months, while other informants were depressed for 6 or 7 months each year. “It starts September, October time. But January, February… February is my absolute worst month. It's like I have no go in me, there's no ray of hope anywhere.” (6)

During the summer when feeling well, the informants “forgot” about the winter depression. When it came back, it was described as hitting a wall or* falling into a black hole and being trapped in the darkness*. “This winter was like hitting a damn wall when the darkness set in. It's pitch black when you wake up, pitch black when you go to work, pitch black when you come home. I get panicky. It feels like someone's pulled a bag down over my head and I can't breathe. I feel completely enclosed in darkness.” (18)

Some of the informants described severe* sleep problems and disturbance of daily rhythms* during the winter period. The problems with sleep often preceded the depressive symptoms. Sleep problems included difficulties going to sleep, maintaining sleep, frequent arousals, and having major difficulties getting up in the morning. Others suffered mainly from hypersomnia; however, sleeping for 10–12 h did not relieve daytime fatigue. “Worst of all is the extreme tiredness, and the feeling that more than anything I want to sleep like a bear until I come out of hibernation in April.” (16) The informants described a feeling that internal and external time was poorly synchronized during the winter period, being more alert in the late evening and extremely tired in the afternoon. “My rhythm is completely at odds with the actual time.” (13)

*A pronounced lack of energy, an overwhelming feeling of tiredness, and inactivity* were major problems for the informants during the winter. The feelings of energy, vitality, and joie de vivre experienced during the summer season flowed away and were lost. This pronounced lack of energy resulted in difficulties initiating and completing ordinary, daily activities. “It's much harder to deal with things and get them done. Everything is a huge effort. I really have to force myself to do things that need doing.” (2) 

*Depressed Mood*. Lowered mood and anxiety increased during the winter. There were severe depressions. “I just cried. It was the only thing I did in principle for a period of two months, every day. Complete hopelessness.” (18) Thoughts came that life was not worth living this way. “I don't want to live, but I don't want to die either. Or I don't have the energy to live, but nor do I want to die. I don't want to live through this winter.” (4) Participants with a milder depression described low energy and increased irritability and sensitivity to stress rather than depressed mood. “I have a stress threshold that is so low, so low. I react really badly to all kinds of stress. And when I react badly to stress I get mad and angry.” (9) These informants were able to continue their work but described a low quality of life during the winter season.

Some participants described clear* seasonal changes in food habits and weight*. During the winter period, informants preferred eating warm, more robust, and “heavy” food (e.g., pasta). In the summertime, the preference was for cold and “lighter” food (e.g., salads). Some experienced craving sweets. Sweets were used as an attempt to increase a low energy level. One informant described a significant seasonal weight gain. “I notice that with my dog. She goes up in weight during the winter. And that's because I don't take her out for exercise. It's a bit awkward because people can see she's become fat. Yes, it's the same for me, although people don't mention it.” (5)

Informants described* a feeling of being “alive” only half of the year*. During the winter, there was a feeling of not being able to participate in life: a feeling of mental hibernation. In the summer, on the other hand, the participants felt they were coming back to normal and coming back to “oneself.” (6) “With the arrival of spring and lighter days, that's when I feel alert, I've got energy, I really enjoy living.” (1)

#### 3.1.2. Consequences for Everyday Life

The informants experienced* difficulties to meet the expectations of everyday life* during the winter season. “It's a source of stress during that entire part of the year, having to perform while not actually having the energy.” (16) There were descriptions of* disturbed relationship with family and friends* during the winter season. It was the inactivity, irritability, and depressed mood that made it difficult for other members of the family. “What happens is that I sleep and don't do anything of my own volition. I have no contact with my friends during those periods. I lie on the sofa and watch TV and don't take the initiative to do the housework. So naturally that affects the entire family.” (5) “What was so difficult was that my depression affected my daughter so much. Finally we ended up at the Child and Adolescent Psychiatry Clinic because her day care center rang the alarm. She was being unruly and playing up.” (18) Informants experienced a* reduced capacity for work*. The lack of initiative was especially difficult for students who missed classes and for entrepreneurs because they were at risk of losing their income. “It was difficult because I didn't have a boss who said that I had to work.” (5)

Some informants experienced* a dilemma because they knew the diagnosis and the treatment were not considered legitimate in the Swedish health care*. “It may be a placebo. Quite honestly, it doesn't matter. If anything has this effect on me which means I don't need pills, then I will happily continue with it.” (6) There was a fear of not being able to receive the treatment in the future because they knew that light rooms had been closed down during recent years and only few were still in use.

#### 3.1.3. Coping Strategies

Some informants used* passive coping strategies like “wait and see” or “hope for the best next year”*. “It's got worse over the years, but I don't want to talk about it very much because I wish it weren't so. Next year it won't happen, that's what one always believes, thinks and wants.” (5) Other informants used more* active coping strategies and tried to maintain physical and outdoor activities, which was considered helpful but difficult*. “I should force myself to go to some sort of keep fit during the winter. But it doesn't happen because I don't have the energy.” (17) Informants with jobs* prioritize work in front of all other activities*. “I cried a huge amount from exhaustion, but I still went to work. Because I was used to going to work even if I didn't feel well.” (7) As the depression got worse during the winter season, the informants refrained from social contacts and recreational activities. “This last winter, I couldn't face meeting up with anyone and had no energy to call anyone. I have quite a large network of friends, but it's like I just hide away, I don't have the energy.” (18) Those informants who could afford it used* travelling abroad* during the late autumn as a way of coping with the winter depression. “A trip around October-November time is almost like going for light treatment.” (9)

For informants with severe depressions, taking* antidepressants* was considered necessary. Some were taking antidepressants semiannually or changed the dose depending on the season. For informants with milder depression, antidepressants were not considered an option. Some had tried medication but stopped because it did not help them or they experienced side effects. “I have tried a lot of things, above all antidepressants and sleeping pills, but I haven't experienced any great improvement as a result. I've had a lot of side-effects which were so worrying that I finally decided that it wasn't worth it.” (13) One informant had tried* psychotherapy* but with no major effect on the seasonal symptoms. “I've had psychodynamic therapy and also CBT. But despite trying hard in therapy, this feeling still came over me. It's like I've understood that it's a matter of something else, something biological.” (12) In addition to travelling south to sunny latitudes and taking antidepressants, only* light room treatment* was considered helpful. “There's not much I can do, there's very little that makes a difference. I haven't found anything. What I think has been most noticeable is the light treatment.” (13)

### 3.2. Patients' Experience of the Light Room Treatment

The analysis of the patients' experience of the light room treatment resulted in three categories and 11 subcategories (Table 3).

#### 3.2.1. Positive Treatment Effects

The informants described the overall treatment effect as* a radical, sudden, and profound transformation*. Metaphors as “awakening,” “illumination,” “coming back to normal and to oneself,” “to climb from the basement up to the roof,” “like a conversion,” or “like becoming a new person” were used. “I felt really good straight after the first ten sessions. I still remember the almost physical relief I felt throughout my body. I was speechless and totally amazed at what had happened. It was as though a yoke had been lifted from my shoulders, a purely physical feeling that I was now free of something.” (9)

The change was described in “energy” terms: a physical sensation of something happening in the brain and with the body and mind feeling electrified and more energetic. “During the first days in the light room I don't notice that much difference. But during the second week, when I sit there for the final half hour, that's when this feeling comes over me. And then when I go out on to the street and it's grey weather, I still feel a sort of charge of energy in my being, or whatever you can call it. I feel it in my legs and body and arms like an electric charge.” (5) “I felt straight away that some sort of change had taken place. I felt something happen inside me… a very distinct change… it feels like a little “ping” in my brain. Something is brought to life, and it happens after the second session of treatment. What I notice is that I'm given energy.” (1) The informants reported* increased feelings of energy and activity*, being able to achieve more during the day. “I came home and set about making dinner and baking bread in the evenings, as opposed to just lying down and crashing out.” (6)

*Mood and thinking improved* following treatment. Anxiety, irritability, and rumination were reduced and feelings of joy increased: “… and it's not as difficult thinking and keeping a lot of thoughts in my head when I'm at work. I feel in a lighter mood, I don't brood quite as much. It feels as though I don't have this cap fixed on to my head the way I do when I get depressed, which prevents me from trying to do things or trying to think differently. I felt as though I became lighter both physically and mentally.” (13) “It's like a weight on my shoulders. Like a bloody millstone round my neck. It was so strange, having had that millstone round my neck and then suddenly having it lifted from me. Despite it being just as dark and miserable outside, it was somehow not as dark and miserable any more. It wasn't as oppressive.” (9)

Informants described that* problems with sleep and daily rhythms improved* following treatment. “Before that I'd had trouble sleeping, I slept an hour or two. But now it's completely different. Now I don't wake up, I sleep all night, and around seven o'clock I wake up of my own accord.” (8) “At this time of year my daily rhythm goes totally haywire, my body doesn't really know when it's time to be awake and when it's time to sleep. I have quite a lot of trouble sleeping; I can lie awake for a long time before getting to sleep at night. And things got better as a result of the light treatment. I then became more awake while I was sitting there. And felt more tired and found it easier to wind down in the evening.” (13) The informants reported that improvement in their sleep and daily rhythm often preceded improvement in other symptoms. Informants reported* positive changes in food habits*, a normalization of eating habits following treatment. “As soon as I have had my light therapy I notice that I have far more energy, and my appetite is restored.” (16)

#### 3.2.2. The Importance of a Good Environment in the Light Room

The treatment was a* calming and relaxing experience* for the informants, albeit some initial feelings of stress or boredom. “The first day I found utterly boring. When I took a book with me to read, and then began to feel it was enjoyable.” (4) Informants stressed* the importance of mutual respect and silence* in the light room. This was especially important at the beginning of the treatment when feeling depressed. Mobile phones were accepted if all sounds were turned off and when not used for talking during the treatment. Other patients talking too much were considered disturbing. There were some problems in relation to* the physical environment* in the light room, for example, bad air quality and the temperature being too high or too low.

#### 3.2.3. Negative Experiences Related to the Light Room Treatment

Some informants experienced* side effects during the first (but not the second) week* of the treatment, for example, mild headaches, increased tiredness, or difficulties going to sleep in the evening. “I got really tired during the first week especially. The second week wasn't quite as bad. But the first week I was completely washed out.” (18) Some informants were disappointed because of* insufficient treatment effects* or because the treatment effect was not the same each year. Some described that the effect lasted only a few days or weeks, which made it necessary to continue the light treatment during the winter. “I think I felt the effect quite quickly and then I had to sustain it to ensure it took hold properly. And my idea is to continue going twice a week.” (13) The* treatment was considered time-consuming* because of travelling time to and from the psychiatric outpatient clinic and for some stressful because of having to get up earlier than usual to participate in the treatment.

## 4. Discussion

This is the first study with a qualitative method investigating patients' experiences of seasonal affective disorder (SAD) and of light room treatment. The overall finding was a description of a profound struggle by the patients to adapt to a recurring situation experienced as inevitable and apparently difficult to escape. The winter depression affected not only the patients' subjective well-being but all important aspects of everyday life, that is, work capacity, recreational activities, and relations with family and friends. There were feelings of being “alive” only half of the year (summer) and feeling like a robot the other half (winter).

In a phenomenological study of the differences between nonseasonal depression and grief, depression was described as a place of darkness, beyond the person's control, and leading to secondary losses [36]. This is similar to the results in the present study. The descriptions of the symptoms are congruent to previous descriptions of symptoms in SAD [2, 37, 38]. Our results are also congruent with studies showing that patients with SAD experience low quality of life during the winter season which changes for the better in the summer [9], after light box treatment [39], and following light room treatment [8].

The effects of the light room treatment were described as a radical and sudden change for the better, usually within the first week. Improvement occurred in all the major symptoms, mood, sleep, daily rhythms, and thinking, energy, activity, and food habits. The light room treatment was described as one of the most important available coping strategies for the patients to improve the situation and regain a sense of control and hope.

The physical and psychological environment was of importance for the experience of the light room treatment. The treatment was described as a calming, positive, and relaxing experience. There were some descriptions of negative experiences; the treatment was considered time-consuming; the treatment effect was sometimes described as insufficient and, at times, other patients in the light room were described as slightly disturbing. There were some initial side effects, but these were experienced as mild and transient. Most patients tolerated the treatment in the light room well, which is similar to findings from treatment studies with light boxes [12, 20, 40, 41].

One finding of special interest is the description of* a “sign*,*” *a signal that functioned as a personal marker for the beginning of the seasonal depression. It was described as a sudden recognition of a thought, a feeling, or a change in behavior and was accompanied by an almost instantaneous reaction of uneasiness or aversion.

Another new finding in this study was the description of* a dilemma* between the personal experience of an effective treatment and the awareness that this treatment is not endorsed (evidence-based) in the Swedish health care system [17]. The patients worried about not being able to receive the treatment in future depressive relapses. Whether the positive treatment was due to a placebo effect or not did not matter much to the patients. The “proof” the patients needed was that of their own experience of renewed energy, better mood, and increased activity.

Another finding was that the deterioration of* sleep and daily rhythms* in the autumn preceded the experience of depressed mood and inactivity. The sleep problems (insomnia and hypersomnia) and delayed circadian rhythms described by the patients in this study are well known in SAD [10]. In a study using 24 h actigraph recordings of patients with SAD, total daytime activity was reduced by almost 1/3 in SAD patients compared with healthy controls. After 4 weeks of LT in that study, there were no differences between groups [42]. The authors concluded that the findings suggested that LT increased blunted activity levels, restored circadian rhythms, and improved sleep in SAD patients. These findings are congruent with how the patients in the present study described how activity, sleep, and daily rhythms improved following light room treatment. There is strong evidence that the human circadian system is entrained by exposure to light and darkness [43]. There is some evidence that patients with SAD become depressed in the winter because of internal circadian misalignment [44, 45]. Researchers try to find a firm association of genetic markers for gene based analysis of circadian genes and seasonal pattern in bipolar disorders as recently reported by Geoffroy et al. [46].

Light and the seasons obviously have strong effects on our biology. But are there also psychological factors associated with seasonal affective disorder? The concept* illness cognitions* is defined as patient's own beliefs about their illness [47]. These cognitions constitute a personal framework for understanding and dealing with illness. There are five dimensions related to illness cognitions: identity (symptoms and diagnosis), perceived cause (biological, psychosocial, or stress), the time line (“how long will it last?”), consequences (physical, emotional, and social) and curability/controllability (“can it be cured?”) [47]. The first part of the results from the present study is a description of illness cognitions in patients with SAD: the perception of symptoms, perceived cause, consequences for everyday life, and coping strategies used. The impact of the different aspects of illness cognitions is only partially known in SAD. In cognitive-behavioral therapy (CBT), dysfunctional cognitions are a focus of the treatment and there is some evidence that CBT is effective for patients with SAD [48–50]. In the present study, however, psychotherapy was not described by the patients as effective for alleviating the seasonal symptoms. This may be an effect of “sample bias,” since only patients who completed light room treatment were included. It is reasonable to assume that some patients with SAD benefit more from CBT, while others benefit more from LT or a combination. Who benefits from the light room treatment (moderators) and what parts of the treatment is effective (mediators) require further investigation.

Is the dark season a stressor for vulnerable individuals? Psychological factors are central to the stress response and coexist with the physiological changes. The* transactional model of stress and coping* describes how individuals deal with stressful experiences [51]. When facing a stressor, the individual evaluates the situation through primary (“is it stressful, harmful?”) and secondary appraisal (“can I cope?”). The primary appraisal includes the perception of personal susceptibility and severity of the stressor. In this study, the informants described a clear perception of personal susceptibility and a strong negative reaction to the early signals of the winter depression (that is, a strong negative primary appraisal). The “secondary appraisal” is the individual's perceived ability to manage the situation, to alter the situation, and/or to handle negative emotions (coping strategies). The patients were very clear in their descriptions of effective means to handle the seasons. For some, antidepressants were necessary; for others they were not considered an option. Physical activity was considered helpful but difficult to achieve during depression. All patients described social withdrawal and reduction of activity as necessary. Travelling abroad to sunny countries was an important coping strategy in addition to light treatment, but not all could afford to travel. Active, approach, and acceptance coping strategies result in better health outcomes for the individual, compared to avoidant or passive strategies [52]. Patients with depression use more avoidant coping strategies compared to the healthy controls [53]. The patients in this study used avoidant strategies during the summer (“wait and see,” “hope for the best”) and active, approach strategies during the winter (travel abroad, take antidepressants, or light room treatment). The results from this study indicate that patients with SAD considered light room treatment one of their most important, active coping strategies. This may be of importance, since a positive association between perception of control and improved well-being and adjustment has been observed across a variety of disorders [52].

### 4.1. Methodological Considerations

The informants provided rich, colorful, and elaborate descriptions of their subjective reality and lived experience, that is, using metaphors and figurative descriptions of symptoms and treatment effects. The sample is well described and presented with extensive background data (Table 1). This, together with the large variation in experiences obtained, facilitates the readers' evaluation of transferability of the results to other contexts [29]. In qualitative analysis, there is no single interpretation of the meaning of a text. Therefore, methods to increase trustworthiness are necessary. Triangulation is a common method to achieve this and was used in the present study; the authors discussed all steps in the analysis until agreement was obtained. Furthermore, four patients who received preventative treatment were included in the study, which contributed to more variation in experiences.

### 4.2. Limitations

A limitation of this study is the inclusion process, which for practical reasons was not based on complete clinical information but on a judgment of the referral in combination with self-report measures of depression. Another limitation is related to side effects. Only patients who completed ≥7 treatments in the light room were included, which is why there may be side effects experienced by patients who discontinued treatment at an early state and were not included in this study.

## 5. Conclusions

The results from this study show that patients with winter depression experience a clear seasonal pattern including deterioration in sleep, daily rhythms, energy level, mood, activity, and cognitive functioning. The disorder affected major aspects of everyday life, with reduced work capacity, social withdrawal, and disturbed relations with family and friends. Light room treatment was described as one of the most effective and personally important coping strategies, with improvement in all the major symptoms and with mild and transient side effects. Considering the 2015 recommendations from the Cochrane Collaboration that the selection of treatment for preventing winter depression should be strongly based on patient preferences, the results of the present study may be used in the clinical evaluation and discussion with the patient and considering possible treatment options. Further studies about the specific individual signs and symptoms profile which mark the beginning of repeated seasonal depressive episodes are called for [54].

## Figures and Tables

**Table 1:** Overview of the informants' background characteristics: sex, civil status, employment, sick leave, medication, previous number of years with winter depression, previous light therapy, preventative treatment or not, and scores on the Montgomery-Åsberg Depression Rating Scale, self-rating version (MADRS-S) before treatment, at posttreatment, and at two weeks after treatment (*N* = 18).

Age (y)	Sex	Civil status^1^	Employment^2^ (%)	Sick leave^3^ (%)	Antidepressants^4^ (Yes/No)	Other current medications^5^ (Nb)	Previous winter depression^6^ (y)	Previous light therapy^7^ (y)	Preventative treatment^8^ (Yes/No)	Pretreatment MADRS-S^8^ (total)	Posttreatment MADRS-S^8^ (total)	2 weeks after treatment MADRS-S^8^ (total)
62	Male	Live apart	100	0	No	0	5-6	2	Yes	0	0	3
64	Female	Live apart	Retired	0	Yes	9	20–30	0	No	24	23	3
24	Male	Married	100	0	No	0	6	0	Yes	10	9	x^9^
60	Female	Married	No	75	Yes	2	8–10	0	No	25	18	3
48	Male	Married	Student	0	Yes	3	29	4	No	15	3	1
28	Female	Married	100	0	No	0	14	0	No	12	4	9
65	Female	Single	Retired	0	No	2	26	0	No	30	0	10
58	Male	Single	Retired	0	Yes	2	24	0	No	30	10	8
41	Female	Married	100	0	No	0	20	5	No	22	7	1
41	Male	Married	100	0	Yes	1	2	0	No	23	8	1
47	Male	Single	Job-seeker	0	Yes	0	10–12	6–8	Yes	0	1	0
37	Female	Married	50	0	Yes	1	20	0	Yes	1	2	x^9^
30	Female	Live apart	100	0	No	0	15	0	No	13	10	4
29	Male	Single	Student	0	Yes	1	13	0	No	16	3	0
44	Male	Married	25	75	Yes	4	15	0	No	20	25	x^9^
41	Female	Single	No	100	Yes	4	24	5-6	No	21	20	7
38	Female	Single	No	75	Yes	3	24	0	No	17	17	x^9^
33	Female	Single	50	0	No	0	4	0	No	14	9	9

^1^Married/cohabitant, single/divorced, or living apart.

^2^Employment full-time 75–100%, part-time ≤50%, job-seeker, retired, or student.

^3^On sick leave full-time 75–100%, part-time <50%, or no sick leave.

^4^Current antidepressive medication.

^5^Other current psychiatric and/or somatic medications.

^6^Informants' retrospective estimate of number of years with winter depression.

^7^Previous experience of bright light treatment (number of years).

^8^The Montgomery-Åsberg Depression Rating Scale, self-rating version (MADRS-S), Svanborg and Åsberg (2001).

^9^Data missing.

**Table 2:** Overview of the results regarding patients' experience of seasonal affective disorder: one main theme, three categories, and 19 subcategories (*N* = 18).

Theme	Categories	Subcategories
Struggling to adapt to the inevitable	A clear seasonal pattern	The shift, early signals
		Discover the pattern
		Fall into the black hole and be trapped in the darkness
		Problems with sleep and daily rhythms
		Lack of energy, overwhelming tiredness, and inactivity
		Depressed mood
		Changes in food habits and weight
		To feel “alive” only half of the year

Struggling to adapt to the inevitable	Consequences for everyday life	Difficulties to meet the expectations of everyday life
		Disturbed relationships with family and friends
		Reduced work capacity
		The dilemma, lack of a legitimate diagnosis and treatment

Struggling to adapt to the inevitable	Coping strategies	Wait and see and hope for the best
		Maintain physical and outdoor activities, helpful but difficult
		Give priority to work and reduce social contacts and recreational activities
		Travel abroad
		Consider the pros and cons of antidepressants
		Psychotherapy
		Light room treatment

**Table 3:** Overview of the results regarding the patients' experience of the light room treatment: three categories and 11 subcategories (*N* = 18).

Categories	Subcategories
Positive treatment effects	A radical and sudden transformation
	Increased energy and activity
	Improvement in mood and thinking
	Improvement in sleep and daily rhythms
	Positive changes in food habits

The importance of a good environment	A calming and relaxing experience
	The need for mutual respect and silence
	The physical environment

Negative experiences	Initial side effects
	Insufficient treatment effects
	Treatment is time-consuming
